# Innovation in Technology Instead of Thinking? Assetization and Its Epistemic Consequences in Academia

**DOI:** 10.1177/01622439221140003

**Published:** 2022-12-05

**Authors:** Ruth Falkenberg, Maximilian Fochler

**Affiliations:** 1 Department of Science and Technology Studies, University of Vienna, Austria; 2 Research Platform Responsible Research and Innovation in Academic Practice, University of Vienna, Austria

**Keywords:** research technologies, assetization, innovation, research practices, epistemic choices

## Abstract

This paper draws on the notion of the asset to better understand the role of innovative research technologies in researchers’ practices and decisions. Faced with both the need to accumulate academic capital to make a living in academia and with many uncertainties about the future, researchers must find ways to anticipate future academic revenues. We illustrate that innovative research technologies provide a suitable means for doing so: First, because they promise productivity through generating interesting data and hence publications. Second, because they allow a signaling of innovativeness in contexts where research is evaluated, even across disciplinary boundaries. As such, enrolling innovative research technologies as assets allows researchers to bridge partly conflicting valuations of productivity and innovativeness they are confronted with. However, the employment of innovative technologies in anticipation of future academic revenues is not always aligned with what researchers value epistemically. Nevertheless, considerations about potential future academic revenues derived from innovative research technologies sometimes seem to override particular epistemic valuations. Illustrating these dynamics, we show that processes of assetization in academia can have significant epistemic consequences which are important to unpack.

## Introduction

Researchers’ decisions about what questions to pursue or how to design a certain study are influenced by a range of considerations. Previous studies have shown, for example, how dominant evaluative logics of contemporary academia (e.g., Fochler et al. 2016; Müller and de Rijcke 2017) or prominent buzzwords influence researchers’ practices and decision-making (Penkler, Felder, and Felt 2020). However, surprisingly little seems to be known about the role of novel technological developments in researchers’ decisions and strategic orientation. This is particularly astonishing given that many scientific disciplines crucially rely on technological devices and infrastructure, which in recent years also tend to become ever “bigger, faster, better” (Davies, Frow, and Leonelli 2013, 386).

Different scholars have examined how novel technological developments “transform sciences” (Hackett 2011, 26) and shape how and what we know about the world. For example, developments in digitalization, high-throughput technologies, and the prominence of big data have led scholars to proclaim a new paradigm of data-driven research (see Leonelli 2012), which fundamentally changes research practices in various scientific fields. Authors have even declared “the end of theory” (Anderson 2008) and suggested that research in the life sciences has become “convenience experimentation,” proceeding in a mode of gathering rather than hypothesis-testing (Krohs 2012, 52). While there is little doubt that novel technologies such as those related to digitalization have important impacts on research practices, analyses of researchers’ decisions to adopt technological innovation within the conditions of the current research system seem to be largely missing. As Lee and Helgesson (2020, 679) note, even critical and reflexive accounts sometimes tend to treat innovative research technologies “as phenomena that in determinist fashion reshape…sciences,” without analyzing the processes in which such technologies are adopted or not.

We therefore ask what role innovative research technologies play for scientists who want to pursue particular epistemic goals but are also situated within the norms, value regimes, and temporalities of the current research landscape, increasingly organized along capitalist cultural logics (Hackett 2014; Fochler 2016). Seeing science and its organization as fundamentally coproduced, we analyze the role innovative technologies play for researchers who need to strategically position themselves and their work as innovative within a competitive academic market and anticipate possibilities to generate future revenues in terms of publications and funding opportunities, when they want to build successful academic careers. Conceptually, we draw from previous accounts of assetization in science (Muniesa et al. 2017; Pinel 2021). We start from the notion that in academia today, characterized by high degrees of competition and uncertainty about the future (Sigl 2016; Fochler and Sigl 2018), the establishment of assets as things “that can be owned or controlled, traded, and capitalized as a revenue stream, often involving the valuation of discounted future earnings in the present” (Birch and Muniesa 2020, 2) plays an essential role for scientists. We suggest that the perspective of the asset is particularly helpful for understanding the role of innovative research technologies in researchers’ practices and decisions.

Our argument draws on in-depth fieldwork with researchers working in the fields of plant breeding and soil microbial ecology, located in Austria as well as in the broader European–US research landscape, with whom we reflected on how and why they chose to employ or not to employ particular innovative technologies. Based thereupon, we argue that innovative technologies often acquire a central role in researchers’ decisions because they can be mobilized as assets from which researchers expect to derive future revenues within the dominant valuation logics of the academic system. Innovative research technologies promise future academic revenues, first, by generating interesting results, and second, by signaling innovativeness beyond a narrow discipline in contexts where research is evaluated. In more theoretical terms, innovative technologies allow researchers to bridge valuations of productivity and innovativeness, both of which are dominant in current academia (Falkenberg 2021). In turn, since innovative research technologies promise the ability to tie into both of these valuation regimes (Fochler et al. 2016), researchers anticipate that enrolling innovative technologies will help in getting published, funded, and acquiring jobs.

Of particular importance to us in this paper is not only the description of this specific assetization dynamic but the analysis of its epistemic consequences. We therefore reflected with our participants on how particular innovative research technologies relate to their epistemic agendas and to what extent these technologies are considered helpful to reach particular epistemic goals. We illustrate that the strategic employment of innovative research technologies as assets that promise future academic revenues is not always in line with what researchers consider epistemically valuable. Nevertheless, our interlocutors described a strong need to employ innovative technologies and, at times, considerations about potential future academic revenues derived from these technologies seemed to override their epistemic valuations.

## The Role of (Innovative) Technologies in Research Practices

Technologies play a crucial role in research practices. Next to a tradition of work on scientific instruments in the history and philosophy of science (e.g., Hacking 1983; van Helden and Hankins 1994; Baird 2004), classical laboratory studies have drawn attention to the centrality of research technologies as “inscription devices” (Latour and Woolgar 1986). Today, it seems needless to say that “inventing machines” is an essential “part of discovering nature” (Traweek 1992, 49). Yet, research technologies matter for researchers’ practices not only due to their epistemic potentialities but also because they fulfill strategic functions for them and research groups. Hackett (2005, 788), for example, has pointed out that the “ensembles of research technologies” a group acquires enables but also limits the experiments they can perform and that technologies thus shape the life course of a research group and position it in the scientific landscape.^1^ Research technologies and associated technological practices are not only crucial to a group’s continuous identity or “signature” (Jacob 1989) but also for opening up new fields of inquiry, creating a competitive advantage over other groups (Hackett 2005).

Despite these important insights, more recent studies from Europe investigating how researchers navigate the hypercompetitive academic landscape hardly focus on the role of research technologies in doing so. We get some pointers to the importance of technology by Rushforth et al. (2019), who argue on the basis of two biomedical research groups in the Netherlands that a group’s research technology ensemble is essential for their options to create portfolios of research lines and hence for tapping into different regimes of worth. Fochler et al. (2016) note, again rather in passing, that junior researchers working in the academic life sciences in Austria assess the value of a research topic with regard to the methods the topic allows them to learn and that they can build upon in their career.

While these studies provide important indications about why researchers—situated within the norms, temporalities, and value regimes of the current academic system—might decide to adopt certain innovative technologies, they hardly tackle this question in all its complexity. In this paper, we put the role of innovative technologies in research practices center stage, asking about both their strategic importance and their role in reaching certain epistemic goals. We suggest that to better understand how innovative technologies figure in researchers’ decisions, we need to attend to the specific cultural and organizational logics of current academia.

## Accumulating Academic Capital and Dealing with Uncertain Academic Futures

The basic contours of a capitalist accumulation logic have already been described in Latour and Woolgar’s (1986) cycle of credibility. In subsequent decades, academia has been increasingly described to be governed by cultural logics of neoliberal capitalism (Mirowski 2011; Hackett 2014; Kleinman 2010; Fochler 2016; Muniesa et al. 2017; Rushforth, Franssen, and Rijcke 2019; Pinel 2021); not only is research often directly entangled with commercial interests but capitalist cultural principles increasingly shape processes of knowledge production from the inside. In the current research landscape—centrally characterized by competition between individuals over scarce resources, and hence, by high degrees of uncertainty (Sigl 2016; Fochler and Sigl 2018)—these dynamics become ever more pervasive. While research can in principle be valued along multiple different “repertoires” or “regimes” of valuation, Burrows (2012) and Fochler et al. (2016), for example, have described the academic sphere as increasingly centered on a regime of valuation focused primarily on productivity and competitiveness, highlighting that scientists’ decisions are often dominated by considerations about the accumulation of academic capital rather than other forms of worth.

Importantly, researchers’ considerations about productivity and the accumulation of academic capital are usually characterized by a pervasive future-orientation (Ylijoki 2010; Müller 2014; Felt 2017). Academics are not only busy with producing value at a certain point in time but also with assessing potential future returns: how to best capitalize on current work (Muniesa et al. 2017), how to compile promising investment portfolios (Rushforth, Franssen, and Rijcke 2019), or how to extract worth by renting out valuable assets (Pinel 2021). The concept of the asset as “something that can be owned or controlled, traded, and capitalized as a revenue stream, often involving the valuation of discounted future earnings in the present” (Birch and Muniesa 2020, 2) is of particular significance in this context. As highlighted by Birch (2020), the capitalization of assets plays an especially important role in sectors characterized by a high degree of uncertainty. Academia, where many researchers constantly need to worry about what comes after the end of the current project or contract, is certainly a case in point here. Indeed, Pinel (2021) and Muniesa et al. (2017) have convincingly illustrated how processes of knowledge production can be understood as centered on establishing and managing assets such as data, databases, scientific expertise, research staff, or scientific results that have value both as resources for further production and as property from which rents can be extracted.

We suggest that innovative research technologies can equally take the form of assets and that this perspective is crucial to understand the central strategic role of acquiring or employing innovative technologies in researchers’ practices.

## Research Technologies as Assets Allowing to Bridge Valuations of Productivity and Innovativeness

Innovative research technologies can be mobilized as assets because they promise future academic revenues, first, through generating interesting results and, second, through signaling innovativeness in contexts where research is evaluated. As such, enrolling innovative technologies allows researchers to anticipate that their work will do justice to two important ways of ascribing worth to research, both to the productivity-oriented regime of valuation described above and to a regime in which research is primarily valued for being innovative and “cutting-edge.” As Falkenberg (2021) has argued elsewhere, this latter regime of valuation plays an increasingly important role in the current research landscape. While valuations of innovativeness are often tightly linked to what is required to remain productive and competitive, scientists are simultaneously confronted with what Hackett (2005, 805) has termed a “paradox of risk:” they need to choose problems that are safe enough to secure output in terms of publications and at the same time they are incentivized to take daring and risky choices that might lead to particularly innovative results and “high gain” (Falkenberg 2021). In order to deal with these tensions, researchers may, for example, add “high risk” work packages to relatively safe projects or pursue more uncertain lines of work along others that promise more stable revenues (Hackett 2005; Rushforth, Franssen, and Rijcke 2019). Yet, previous studies have also described how considerations about safe productivity within given time frames often predominate and induce researchers to take more conservative epistemic decisions, especially when they lack “protected spaces” in terms of time and resources, and must adapt to standardized project frameworks (Laudel 2006; Laudel and Gläser 2014; Fochler et al. 2016; Franssen et al. 2018; Whitley, Gläser, and Laudel 2018).

We argue that innovative research technologies provide researchers with another means to deal with these tensions because they promise both productivity and the articulation of research as innovative. This resonates with earlier science and technology studies (STS) work such as Fujimura’s (1988) account of scientific bandwagons, where she illustrated how novel research technologies—once established to a certain degree—not only allow for productive work and the construction of “do-able problems” but also signal innovation and “sexiness.” Particularly important, innovative research technologies can even help articulate innovativeness across disciplinary boundaries. As described by Knorr-Cetina (1981, 57), when an approach to a problem in one field is transferred to a different problem in another field, this “unrealized solution” is perceived as something innovative but simultaneously holds a certain “promise of success” and productivity precisely because it has already proven to work in a different context. The potential to articulate innovativeness across disciplinary boundaries is significant because what comes to be understood as innovative is generally context-dependent and differs between disciplines (Guetzkow, Lamont, and Mallard 2004; Barlösius 2018; Rammert et al. 2018; Pickersgill 2019). This can pose problems to researchers when they, for example, need to articulate the innovative and exciting character of their work to broad interdisciplinary review panels.

By promising both the articulation of innovativeness across disciplines and productivity, innovative research technologies to some degree help make “the uncertainty inherent in innovation understandable and calculable” (Birch and Muniesa 2020, 3). They take the form of assets that allow researchers to anticipate the generation of future academic revenues in terms of publications or successful funding and job applications. As such, innovative research technologies have a crucial enabling function in navigating uncertain academic futures. Yet, we emphasize that it is indispensable to also understand how these dynamics relate to what researchers value epistemically, meaning what they consider as most suited for reaching particular epistemic goals. Indeed, the employment of innovative research technologies as assets that promise future academic revenues is not always aligned with such epistemic valuations. Nevertheless, our interlocutors often described a strong need to employ innovative technologies and, at times, considerations about potential future academic revenues derived from these seemed to override particular epistemic valuations. As such, processes of assetization are not neutral but come to reshape epistemic practices in impactful ways.

## Case and Methods

Our analysis is based on material produced in the project Valuing, Being, Knowing. Understanding the Entanglements of Valuation Practices and Subjectification Processes in Life Science Research, where we are interested in how researchers make decisions within the multidimensional value regimes, norms, and temporalities of current academia.^2^ Empirically, the project is based on a close collaboration with three research groups from the crop and soil sciences based at two different Austrian universities. Both universities share a focus on excellence in basic research, but one is more explicitly oriented toward producing knowledge of agricultural and environmental relevance, while the other is a more comprehensive university with a broader mission in research and teaching. Participants gave written consent to their participation in the overall project, including the participation in the different empirical activities. Between October 2019 and May 2020, we conducted a total of forty-two interviews with researchers of various career stages from the three groups and occasionally observed their experimental work and lab meetings. These observations and the qualitative, semi-structured interviews aimed at acquiring an in-depth understanding of the researchers’ epistemic agendas and how these and their careers have developed over time. The interviews also focused on mapping the various aspects that matter to them in making decisions in their research. Furthermore, between June and August 2020, we conducted three focus groups, in total with twenty researchers from the three research groups, again across career stages, though not mixed across groups. The focus groups centered on meanings and valuations of innovativeness in the current research landscape (see Falkenberg [2021] for a more detailed description of the conceptualization of the group discussions). While our thinking and analysis are based on the entirety of these data, in the following, we mostly draw from our work with two of the groups, as it would go beyond the scope of this paper to provide a detailed account of the relations between innovative research technologies and the groups’ epistemic agendas for all three of them.

The first group we focus on works in the field of plant breeding. In addition to the material described above, we also participated as observers in a three-day conference organized by the group in February 2020 and in online meetings of a larger project consortium the group is involved in. Both the conference and the consortium meetings centered on innovative research technologies in different ways. The other group this paper focuses on works in soil microbial ecology. Since our engagements with the group as described above indicated soil microbial ecology to be a particularly interesting case regarding the role of innovative technologies in research practices, we decided to further inquire into these dynamics in the field more broadly. Between March and June 2021, we conducted an additional sixteen interviews via video call with prominent researchers from the international soil microbial ecology community. The respondents were located on different continents, mostly in the Global North. Written or verbal consent was given for every interview. Furthermore, we organized a follow-up discussion with six of the interviewed researchers and some participants engaged in additional e-mail conversations with us. Our engagement with the field of soil microbial ecology and our analysis of the dynamics we observed are described in more detail in another paper (Falkenberg et al., forthcoming) but equally provided very relevant material for the present analysis.

As visible from our descriptions above, the analysis in this paper developed in a longer iterative process of doing fieldwork, empirical analysis, and feedback sessions with the participating groups. This process was guided by a grounded theory sensibility (Charmaz 2006) and involved several rounds of coding. Feedback by researchers from the participating groups was important in developing the argument, and included feedback to draft versions of this paper, which we are grateful for.

The close engagement with the research groups provided us with a very deep understanding of their work. This allows us to provide textured accounts that speak to the challenge of identifying the epistemic consequences of the assetization of research technologies. In our work with the groups, a constant focus was to collaboratively reflect how the experiences of the groups, situated in two specific research fields, relate to broader trends in contemporary academia. Parts of the fieldwork, such as the interviews with international researchers, served to validate these insights beyond the Austrian context. Nevertheless, the empirical analysis in this paper is based on the situated experiences of researchers in the fields of plant breeding and soil microbial ecology. Our aim in this paper, then, is to build a conceptual argument that sketches some more generalizable trends and conclusions that arise from these situated perspectives. We hope that later research will test, contextualize, and nuance our findings further.

## Enrolling Technologies as Assets, Anticipating Future Academic Revenues

In the following, we first draw from different empirical examples to sketch how innovative research technologies are enrolled as assets from which researchers (expect to) derive future academic revenues when it comes to producing publications and attaining jobs or funding.

### Capitalizing on Innovative Digital Technologies in Plant Breeding Research

The plant breeding researchers we worked with aim to identify crops resistant to different diseases and to understand the genomic basis of these resistances. Traditionally, plant breeding has relied on the phenotypic selection of plants, yet research in the field today increasingly draws from different computational and digital technologies. In recent years, the group we collaborate with has been integrating such technologies into their research portfolio as valuable assets from which they plan to derive future academic revenues.

For example, the researchers now not only employ phenotypic or marker-assisted selection but also genomic selection based on next-generation sequencing and novel computational technologies, allowing them to predict characteristics of plants using simulated data. Establishing this set of technologies as a valuable asset for the group involved attaining the relevant computational infrastructures but also hiring a postdoc with respective bioinformatic expertise—“our young, very active lad, who is really into this stuff” (R1)—who helped the group integrate genomic selection technologies into their work. Employing genomic selection as one of the up-and-coming approaches in the field allows the researchers to anticipate that their work will do well in future reviews for funding and publication because others will recognize this approach as innovative and “sexy” (R53).

Another set of innovative technologies the group was about to acquire were digital high-throughput technologies such as camera-carrying drones, which replace the manual evaluation of plant traits. When the principal investigator (PI) of the group explained this so-called phenomics approach to us, they wondered, “can I fly with drones over [the field] and…from any aerial pictures…where all kinds of things can be measured, interpret these measurements in a way that it becomes interesting for agriculture or breeding?” (R1). As we later elaborate, this question was not necessarily answered in a positive manner for the specific research practices of the group, yet the PI was convinced that these technologies would constitute an extremely valuable asset which they can capitalize on in future funding applications, and also in publications.

Explaining why they considered the acquisition of this set of technologies valuable, the PI was rather explicit about the promised future revenues in comparison to the approach the group employs at the moment, noting that “it’s good, because if for future projects, we can write: we do phenotyping with imaging systems, and this is the most innovative method at the moment, this will be a plus. But if we write, we run kilometers and kilometers every week across our fields and use our eyes—old fashioned” (R1). Moreover, the phenomics technologies not only seemed to be viewed as a valuable asset by the research group itself but also by the university the group is part of. As the PI explained, when they suggested to the university that “we have to do something about modern phenomics” (R1), the university granted a substantial amount of money for acquiring those technologies. For the university, this set of technologies takes the form of an asset that promises future revenues not only because it can allow research groups to successfully acquire third-party funding, but also because it raises the university’s prestige as part of a broader digitalization agenda.

Importantly, the plant breeding group is also constantly engaged in the active cultivation and management of these technological assets. In recent years, researchers from the group have been involved in several workshops on genomic selection and during our collaboration they hosted an international symposium on digital technologies in plant breeding. Thus, the group’s researchers continuously worked to emphasize the important and innovative character of these technologies within the wider plant breeding research community, and to establish themselves as an important player in the field of “digital breeding”—making the group attractive to other researchers who might come work at the department in the future. Overall, these practices then allow the group to maintain or enlarge the value of their technological assets.

### Enrolling Innovative Technologies to Make a Successful Career in Soil Microbial Ecology

Researchers often described soil microbial ecology as being, or having been, strongly driven by innovations in research technologies. After the field had been limited to the difficult and time-consuming cultivation of microbes in the laboratory, subsequent technological advances brought about various new possibilities for investigating soil microbial communities—being compared to “the invention of glasses” (R38) and promising to overcome what was previously seen as a “black wall” (R12). Against this background, many soil microbial ecologists described innovative research technologies as central to their work and to serve an indispensable role for getting published or to receive funding. Moreover, conversations with soil microbial ecologists were particularly illustrative of the central role innovative technologies as assets can play in the career-related decisions of individual researchers.

The PI of the group we collaborate with, for example, described considerations about learning to work with a particular mass spectrometric technology for analyzing single cell functioning—an approach that was seen as extremely “cool” and innovative within the field—as key to their decision to apply for a particular position in their postdoc phase. As they explained:

the drive for what I did [in the postdoc] was more technological. There was this NanoSIMS, that was really really new…and I could use it there because it was one of the labs who did that from the beginning. So there was this realization, this is a really good method, it is really good to know how this works for everything you want to do after. (R12)

They thus described the choice for this particular position as explicitly driven by considerations about the future benefits of learning to work with this technology in building a successful career. As we learned, this bet played out well indeed: the technology proved to be an extremely valuable asset they could capitalize on when applying for their next position. They went on to describe that “in the end, because of this technology I got the offer to come here [to their current position]” (R12).

Paralleling the dynamic this researcher described with regard to the novel mass spectrometric technology, other soil microbial ecologists explained that acquiring skills in high throughput-sequencing and especially metagenomics technologies—another “hyped” approach in their field—had played similar strategic roles for them. Various soil microbial ecologists even described a certain pressure for young researchers to learn to work with such approaches to make a career in the field. As a senior researcher from our international interview sample recounted:

I’ve had a very good student…started work on a project and she said…“there’s no high-throughput sequencing in this project, can we do something.” I said “why,” and she said, “I know there’s no need to do it but every interview I had before this one, they wanted expertise in high-throughput sequencing.” So, she wanted to do that to possibly get the next job. (R49)

Along these lines, participants had the impression that part of the motivation for decisions to work with innovative research technologies was “not the science,” but rather “what people need to make their careers” (R49).

In sum, innovative technologies seem to play an important role in these researchers’ decisions, taking the form of assets from which researchers anticipate to derive future value when it comes to acquiring funding, job positions, or getting published. Yet, why and how precisely do innovative research technologies allow researchers to anticipate future revenues in the present?

## Research Technologies as Promising Productivity and Signaling Innovativeness

In our discussions, participants sometimes explicitly spelled out why they saw innovative research technologies to allow them to anticipate future academic revenues. First, they often highlighted that innovative technologies promise a revenue stream in terms of interesting data that can be converted into publications, hence ensuring productivity. As one senior researcher from the international sample put it, “techniques generate data that are seen as necessary for publications, which are the major tangible deliverable…new techniques offer greater potential for generating data and measurements that have not been made before” (R49). Some researchers also described how employing novel research technologies can allow for new measurements, insights, and publications even without necessarily asking completely new questions. A postdoc from one of the groups we collaborate with noted that “sometimes you can address old questions and things which have been done before, but with a new method” (R13). Or, as another researcher from our international sample highlighted, “if you have a lot of money, you can generate lots of data with the fanciest techniques and then you of course get into the top journals…and I sometimes have to smile because somehow, we did similar experiments twenty years ago…of course with much simpler methods, and less data” (R39). Such promises for revenues in terms of publications are then important for researchers in and of themselves, but also because they can function as resources to be capitalized on in future applications for funding or job positions.

Second, innovative technologies also promise future academic revenue because they play an important role in signaling innovativeness—even across different disciplines—in contexts where research is evaluated. This is important because in order to get funded, researchers increasingly need to justify the innovative character of their work—both to their closest peers and to reviewers from different disciplinary backgrounds. For example, the Austrian groups we collaborate with mainly rely on the highly underfunded and thus highly competitive Austrian Science Fund, as well as on European funding schemes. In both cases, participants anticipated that their proposals would not only be assessed by disciplinary peers in a narrow sense: “these reviewers of your proposals, nine times out of ten, they are not per se people from your immediate circle, of having similar interests” (R18). In such contexts, participants described it as potentially difficult to argue for the innovativeness of rather niche-specific research questions. In contrast, our interlocutors from both the local research groups and the international sample suggested that innovative technologies are more often known and applied across different disciplines and can thus be more easily recognized as innovative beyond one discipline. As one researcher put it:

it’s easier to argue that you’re innovative with a novel technology, because these are often more interdisciplinary compared to questions. I could come up with a really cool innovative question about the organism I study, but [another researcher] might think that’s completely boring. But she might be interested in the method. (R18)

Along these lines, another participant noted that generally “scientists and funders can get excited more easily about new technology” (R52), which makes it more likely that work using innovative technologies receives funding or gets published.

Overall, innovative technologies can thus function as appealing “unrealized solution[s]” (Knorr-Cetina 1981, 57) that promise future revenue by generating interesting data and by signaling innovativeness. Enrolling innovative research technologies as assets allows researchers to bridge partly conflicting valuations of productivity and innovativeness they are confronted with in the current research landscape (Falkenberg 2021)—thereby promising future revenues in terms of publications, funding, and jobs. As researchers pointed out, innovative technologies do in this regard differ from novel and exciting research questions, which cannot take the form of assets that promise future revenue streams so easily. Very innovative questions are often characterized by high uncertainty and as such, they may even render future revenues unpredictable rather than controllable. While an innovative question can turn out to be very productive, delivering “high gain,” it can also carry a “high risk” of failure. In contrast, innovative research technologies may even make a publication or a project (appear) innovative independent from its content, thus potentially helping to circumvent the tensions between doing research that is both innovative and safely productive.

## Academic Revenues versus Epistemic Worth? The Value of Innovative Research Technologies for Knowledge Production

So far, we have established that innovative research technologies can be enrolled as assets that promise to bridge requirements to be both productive and innovative and that thereby help researchers anticipate future academic revenues. We have shown that these anticipations often seem to play an important role in researchers’ decision-making processes. Yet, we argue that it is indispensable to also attend to the epistemic dimension entangled in these dynamics. Are considerations about employing innovative research technologies in anticipation of future academic revenues aligned with what researchers consider valuable from an epistemic perspective? While our interlocutors generally considered the employment of innovative technologies as important to their epistemic agendas, they were not necessarily and always convinced of the epistemic worth of such technologies, and in some instances, considerations about the future academic revenues that such technologies promise seemed to be in conflict with particular epistemic valuations.

### Not All Innovative Research Technologies Are Epistemically Useful

To begin with, the case of the plant breeding group and their acquisition of different digital research technologies indicates that while researchers may consider *some* innovative technologies as epistemically useful, they may not see others to provide the same epistemic advantage over other, potentially older technologies. Nevertheless, researchers perceived a general need to use innovative research technologies to derive future academic revenues from them.

On the one hand, the plant breeding researchers were appreciative of the genomic selection technologies they acquired and considered these to enrich their research portfolio. In contrast to phenotypic selection (relatively time-intensive), and marker-assisted selection (mainly feasible for selecting simple traits where major genetic loci have large effects), genomic selection allows researchers to select complex quantitative resistance traits in a much faster manner, and to better achieve their epistemic goals. In this case, there is a good alignment between the strategic use of these technologies as assets which promise future academic revenues such as successful funding applications, and their epistemic value in knowledge production.

On the other hand, researchers from the group doubted the epistemic value of camera-carrying drones as a replacement for the manual evaluation of plants—another technological asset they acquired. The PI of the group noted:

I’m very skeptical that this gets us somewhere…but it’s so fancy, we have to do it, although we know from our daily experience, if we walk through our fields and we use our imaging system that we have built in here [points to the eyes], we are really efficient, and I doubt we will be more efficient with the camera flying on the drone. (R1)

This researcher and their colleagues questioned whether innovative technologies for high-throughput phenotyping would be of much use in their particular situation or that they would outperform rather mundane approaches such as manually and visually assessing plant traits. A postdoc colleague of the researcher cited above remarked that innovative phenomics approaches could create new problems and unnecessarily complicate research processes: “now someone has to spend a bunch of time figuring out, looking at the data, how to analyze it, how to store it—I mean it’s huge, so is it really necessary when we could just do visual phenotyping?” (R24).

In this case, epistemic valuations and assessments of potential academic revenues were thus rather in conflict. Still, it seemed that the possible future value of these technologies in getting funded or published could sometimes weigh stronger than researchers’ epistemic considerations. When a postdoc from the plant breeding group wondered about the camera-carrying drones for high-throughput analysis of plant traits, “do we really need all these fancy methods, apart for getting a grant” (R4), the PI laughingly noted that “this is good enough a reason” (R1). This interaction vividly illustrated how considerations about the future academic benefit of a certain technology taking the asset form can sometimes even override particular epistemic considerations.

### Appreciating Innovative Technologies, but Not for All Kinds of Problems

Turning to the soil microbial ecologists, we can equally find ambivalent epistemic valuations of innovative research technologies. Again, as noted before, innovation in technology was seen as essential to advance their field and as immensely valuable in epistemic terms. Yet, the case of soil microbial ecology illustrates that the selfsame innovative technologies, while considered useful as such, might not be seen as epistemically valuable in every instance and for all kinds of problems—which can, again, be in tension with the perception of a rather unconditional need to employ such technologies to succeed in the academic landscape.

As hinted earlier, metagenomics, a technology once considered to be highly innovative and exciting within the field, is now seen as ambivalent with regard to its epistemic value. Metagenomics is considered to have brought essential advances to soil microbial ecology because it allowed researchers to sequence the DNA of entire microbial communities directly from environmental samples instead of being limited to the tedious cultivation of individual microbial strains in the lab. As such, it enabled extremely valuable insights into the composition and potential functioning of soil microbiomes. However, researchers noted that, despite this, metagenomics would be a limited approach when it comes to gaining insights into the actual functions of soil microbial communities within ecosystems. To answer such questions that soil microbial ecologists now consider interesting and relevant, they found it important to combine metagenomics with a broader set of approaches, including older, less “sexy” (R53) research technologies, which can still be epistemically useful although they have become disregarded as “old-fashioned” (R33) following the rise of metagenomics and other omics technologies. For example, researchers considered it valuable to go back to approaches such as cultivating microbes in the lab, which had almost become “a science getting extinct” (R12) and was only recently recognized to still be necessary for particular kinds of knowledge production.

However, soil microbial ecologists sometimes saw a tendency in their field to resort to innovative research technologies such as metagenomics even when they might not be best suited in epistemic terms. Reflecting on a recent paper, one postdoc elaborated:

the metagenomics did not tell anything—you have a lot of data, but the conclusions were so general because the authors were so lost in this mass of data…and that was published and the technology was new, but the insights were maybe not bigger than those from cultivation or functional gene studies. (R13)

Participants even had the impression that it could sometimes be hard to get funded or published without including such cutting-edge technologies and that “when you write something and you use a rather old method, you wonder, is this going to fly or will they tell you, this is completely outdated, even if it’s still the best method for this question” (R12). Overall, soil microbial ecologists felt that the employment of innovative research technologies such as metagenomics for productivity and career-related reasons had become so normative that it led to a certain epistemic distraction from the actually important ecological questions in their field.

### Focusing on Innovative Research Questions rather than Technologies

Thus, there are instances where researchers’ epistemic valuations of innovative technologies and considerations about the future academic revenues these technologies promise go well together, and others where they are not so well aligned. Researchers across groups as well as in the international sample, however, frequently pointed out that they would consider it rather problematic if innovative technologies are primarily employed because they promise academic revenues in terms of publication, funding, or job positions, rather than because they are well suited to answering certain research questions.

Participants found it very important that science is ultimately guided by questions rather than by particular technologies. As one of our locally collaborating PIs noted, “I really think the biggest innovation first lies in the questions you ask, then the techniques come in” (R7). Indeed, various interlocutors stressed that they would see the appeal, innovativeness, and the overall value of a research endeavor not as being determined by whether it employs an innovative technology or an older one. Instead, as one PhD from the same group demanded, technologies should be selected according to whether they allow to answer particular questions rather than employing innovative technologies “just because you have them” (R9) and they might be strategically valuable, while only subsequently “looking for problems to apply [them]” (R9). Yet, despite their strong sense that research should be guided by questions rather than innovative technologies, researchers voiced concerns that the current dynamic was the exact opposite.

## Conclusion

In this paper, we have argued that the notion of the asset is helpful to better understand the central role that innovative research technologies acquire in researchers’ practices within the valuation logics of the current academic system, and we have been raising questions about the epistemic consequences of this specific assetization process. Confronted with the need to accumulate academic capital to make a living in academia (Fochler 2016), and with many uncertainties about the future (Sigl 2016), researchers constantly (have to) assess what is likely to generate future academic revenues. Drawing on extensive fieldwork with researchers from the fields of plant breeding and soil microbial ecology based in Austria as well as in the broader European–US research landscape, we have argued that innovative research technologies promise such future academic revenues, first, in the form of interesting results and hence productivity and, second, because they allow a signaling of innovativeness, even across disciplinary boundaries, in contexts where research is evaluated. As such, enrolling innovative research technologies as assets allows researchers to bridge partly conflicting valuations of productivity and innovativeness they are confronted with in the current research landscape (Hackett 2005; Falkenberg 2021). Through promising the ability to tie into both these regimes of valuation, innovative technologies allow researchers to anticipate future academic revenues in terms of publications, funding and job applications. Yet, while having an important enabling function, the enrollment of innovative research technologies in anticipation of future academic revenues is not always aligned with what researchers value epistemically. Nevertheless, participants described a strong need to employ innovative technologies and, at times, their considerations about the potential future academic revenues derived from such technologies even seemed to weigh stronger than particular epistemic valuations.

To better understand the role of innovative research technologies in research practices, and to avoid latent techno-determinist tendencies that see novel technological developments as unavoidably driving and shaping epistemic processes (Lee and Helgesson 2020), it is necessary to scrutinize how and why scientists decide to adopt or not adopt innovative research technologies. How do these decisions relate to their epistemic agendas and how do they relate to the value orders of neoliberal regimes of research governance?

We see this paper as a contribution to discussions about the epistemic consequences of current research governance and the increasing presence of capitalist cultural logics in academia on different levels, adding to existing work on processes of assetization in science. While other STS scholars have convincingly applied the notion of assetization to the academic sphere (Muniesa et al. 2017; Pinel 2021), previous studies have mostly stopped at showing the presence of such dynamics. Our analysis, however, underlines that the notions of the asset and assetization can be useful conceptual tools for analyzing not only changing dynamics of valuation in research practices but also, more specifically, intertwinements between valuation, anticipation, and epistemic decision-making. While innovative research technologies can take the form of assets that allow researchers to bridge tensions between the valuation regimes of productivity and of innovativeness, this often comes at the expense of particular epistemic valuations. In order to understand this dynamic, it is important to consider that not all things can be made assets equally well. Innovative research technologies can be mobilized as assets that promise future revenues more easily than, for example, innovative research questions.^3^ Very innovative research questions are often characterized by a high degree of uncertainty and may thus render future revenues unpredictable rather than controllable. In the current hypercompetitive research landscape, characterized by existential uncertainties about the future, researchers may thus at times understandably prioritize innovative technologies in their decisions.

As such, processes of assetization in academia are not neutral and can reshape epistemic practices in impactful ways. If researchers feel a strong need to enroll innovative research technologies out of strategic considerations, even in cases where this is not entirely aligned with their epistemic aims, this creates considerable tensions for researchers who consider it relevant to work on questions that are not in line with recent technological bandwagons. Moreover, as we have illustrated by drawing on the case of soil microbial ecology, if research practices become increasingly centered on the employment of innovative technologies, this can lead to a displacement of other less “sexy” approaches that might still be of important epistemic value. It might also, more generally, entail a stratification of research fields around technological hypes.

Our analysis also adds important insights to discussions about innovation in science and frequently voiced worries that innovative research is constrained as a consequence of neoliberal regimes of research governance and pressures to adapt to standardized project frameworks (Laudel 2006; Laudel and Gläser 2014; Fochler et al. 2016; Franssen et al. 2018; Whitley, Gläser, and Laudel 2018). Our case study of researchers in plant breeding and soil microbial ecology suggests that it is necessary to add more empirical specificity to such concerns and to investigate how innovativeness in research may not only be hampered altogether but also how *what comes to counts as innovative* is shifting within the current scientific landscape. As innovative technologies acquire such a central strategic role in researchers’ practices—one that innovative questions cannot fulfill—several participants voiced concerns that “innovation in technology” sometimes overrides or even replaces “innovation in thinking.” These tendencies share obvious parallels with broader sociopolitical pro-innovation biases (Sveiby, Gripenberg, and Segercrantz 2012; Godin and Vinck 2017), where innovation is almost exclusively conceived of as technological and industrial innovation at the expense of, for example, social innovation. The central role that innovative technologies acquire in research practices may thus also be seen as coproduced with these wider societal beliefs in technological innovation as a primary problem-solving strategy.

While these tendencies require further empirical investigation, the trend we have observed here is concerning for both the social and epistemic development of science. If researchers’ decisions are overly driven by possibilities for working with innovative technologies in anticipation of future academic revenues and career-related benefits, this has significant epistemic consequences. In the long run, these dynamics may polarize science in favor of technology-intensive research endeavors at the expense of potentially equally innovative and relevant, yet less technologically attractive ones.

## Notes

1. Hackett (2011) has noted that “[t]echnologies are not merely tools or techniques, but entail a constellation of methods, materials, interpretations, understandings, conventions, skills, theories, and social relationships that collectively constitute a socio-technical system or ensemble” (p. 28). When using the term research technology, we follow this definition, aware of this complex embedding of technologies with methodological approaches, established practices, and theoretical frameworks.

2. In the project, we work together with Lisa Sigl, and most of the material described below has been gathered with her help.

3. Certainly, it is not because of any inherent qualities that some things can be better enrolled as assets than others, but because of particular contextual conditions. While, for example, data can often be well assetized in the current scientific system, this becomes less easily possible with open science approaches that demand openly sharing all kinds of data sets.
